# Mild Malformation of Cortical Development With Oligodendroglial Hyperplasia and Epilepsy: A Systematic Review

**DOI:** 10.1212/NXG.0000000000200240

**Published:** 2025-01-31

**Authors:** Yixin Zhan, Shijia Chen, Zhenghan Jin, Jiping Zhou, Yin-Xi Zhang, Qun Hou, Yi Wang, Guoqing Zheng, Yang Zheng

**Affiliations:** 1 Department of Neurology, The First Affiliated Hospital of Zhejiang Chinese Medical University, Hangzhou, China;; 2 Key Laboratory of Neuropharmacology and Translational Medicine of Zhejiang Province, School of Pharmaceutical Sciences, Zhejiang Chinese Medical University, Hangzhou, China;; 3 Department of Neurology, Wayne State University/Detroit Medical Center, Detroit, MI; and; 4 Department of Neurology, Second Affiliated Hospital, Zhejiang University School of Medicine, Hangzhou, China.

## Abstract

**Background and Objectives:**

Mild malformation of cortical development with oligodendroglial hyperplasia and epilepsy (MOGHE) is a newly described rare entity of drug-resistant epilepsy, with a wide spectrum of presentations. We aim to describe the diagnostic features and prognosis of MOGHE in a large cohort.

**Methods:**

We performed a systematic review preregistered on PROSPERO (CRD42023472978), in accordance with the Preferred Reporting Items for Systematic Reviews and Meta-Analyses statement. We searched PubMed, Embase, Scopus, and ScienceDirect between database inception and November 30, 2023, for all published studies on MOGHE. Inclusion criteria were a histopathologic diagnosis of MOGHE. The risk of bias was analyzed with a standardized tool specifically for case reports and case series. The demographic, clinical, EEG, neuroimaging, genetic, and neuropathologic features; treatments; and prognosis were extracted and analyzed. Subgroup analysis was performed with the age at onset and *SLC35A2* variant status.

**Results:**

A total of 163 patients with MOGHE from 18 studies were included in the analysis. The median age at seizure onset was 1.2 years, and 103 were male. Ninety-five patients presented with unilobed lesions. Ninety-nine had lesions in the frontal lobe. A total of 101 patients achieved a favorable surgical outcome. Patients with an onset before 10 years were more likely to present with epileptic spasms, the West syndrome, a circumscribed pattern of interictal EEG, intellectual disabilities, and a better seizure outcome, compared with those with an onset age 10 years and older. Forty-five patients (72.6%) were *SLC35A2*-positive. Patients harboring the *SLC35A2* variants were more likely to present as Lennox-Gastaut syndrome, when compared with those who were *SLC35A2*-negative.

**Discussion:**

MOGHE is a distinct entity of drug-resistant epilepsy associated with *SLC35A2* variants, characterized by age-dependent phenotypes. The study emphasizes the clinical pearls indicative of the rare disease, which may facilitate early recognition and appropriate selection of treatments. The included studies were case reports or series, which were mainly limited by selection and reporting biases.

## Introduction

Mild malformation of cortical development (MCD) with oligodendroglial hyperplasia and epilepsy (MOGHE) is a newly described histopathologic entity of MCD. The presence of oligodendroglial hyperplasia was first reported by Burger in patients who underwent epilepsy surgery.^1^ Since then, MRI features of cases with similar pathologic findings have been described under the term proliferative oligodendroglial hyperlasia in epilepsy.^2^ It was not until 2017 that the term MOGHE was coined to describe the entity based on patients with drug-resistant focal epilepsy but histologically “nonlesional” pathologies. The distinctive histologic findings of marked proliferation of oligodendroglial cells in the white matter and deep cortical layers constituted the defining feature of MOGHE.^3^ Despite its rarity, MOGHE has been increasingly recognized as a cause of drug-resistant epilepsy.

The presentations of MOGHE are highly variable, including but not limited to epileptic spasms (ESs), tonic or clonic seizures, hyperkinetic seizures, and cognitive impairment.^4-6^ Owing to the overlap in phenotypes, MOGHE can be easily misdiagnosed as focal cortical dysplasia or epileptic encephalopathies before surgeries.^7^ However, unlike other developmental causes of epilepsy, the surgical prognosis is unexpectedly favorable in some patients despite the seemingly widespread seizure network.^4-6,8,9^ Furthermore, MOGHE is also strongly associated with *SLC35A2* variants. This link was first established in a cohort of patients with MOGHE and through a consensus histopathology and genetic trial.^4,10^ Variation in the *SLC35A2* gene may disrupt galactose metabolism,^11^ and pilot studies have suggested that galactose supplementation could be an effective targeted therapy for patients with MOGHE.^12^ Those features distinguish MOGHE as a unique entity, warranting early recognition and appropriate treatment.

The current knowledge of the demographic, clinical, and prognostic features of MOGHE is mostly based on reports of cases or single-center studies. Most studies focused on specific aspects of MOGHE or included either pediatric or adult patients, which may explain the large heterogeneity in the findings. The full spectrum of clinical and prognostic features of MOGHE across age groups remains uncharacterized.

In this systematic review, we pooled individual patient data from reports or studies of MOGHE from multiple centers with a standardized methodology. We aimed to describe, in a large-scale cohort, the demographic, clinical, neuroimaging, EEG, genetic, and neuropathologic features and prognosis of the novel treatable entity. We also aimed to identify potential subgroups according to the age at onset and the *SLC35A2* variant.

## Methods

### Search Strategy

Two reviewers (Y.Z., S.C.) independently searched the PubMed, Scopus, Embase, and ScienceDirect (eMethods). A hand-search through both backward and forward citation tracking of the bibliography of the included record was completed to further identify eligible studies. There is no restriction on the language.

### Eligibility Criteria

Articles published between database inception and November 30, 2023, were considered. Inclusion criteria were a histopathologic diagnosis of MOGHE.^13^ Exclusion criteria were as follows: (1) animal studies; (2) conference abstracts, reviews, commentaries, and preprints, which have either limited data or not been peer-reviewed; (3) treatments including thermal ablation or vagus nerve stimulation, which prevent and interfere with acquisition of pathologic results; (4) studies reporting only general descriptions at the study level without individual patient data; (5) duplicate data of previously published studies. Two neurologists independently evaluated studies (Y.Z., S.C.) before inclusion. In case of disagreement, a third reviewer (G.Z., or Y.Z.) was consulted.

### Data Extraction

For all patients, 2 reviewers (Y.Z., S.C.) independently collected information on the sex, age at onset, age at surgery, epilepsy duration, lesion location, semiology, cognitive or behavioral comorbidities, brain MRI, fluorodeoxyglucose-positron emission tomography (FDG-PET), EEG, histopathology, surgery types, genetic findings, treatment, and outcomes from article text, tables, and figures when available. For cases with incomplete information, missing data were removed and only the available data were recorded and analyzed. A single data set with the abovementioned variables was generated on mutual agreement.

The criteria used to define drug-resistant epilepsy in this study were as follows: (1) drug-resistant epilepsy specifically reported; (2) if not specified, patients with 2 or more antiseizure medications (ASMs) were considered drug-resistant.^14^

If the cases had clearly reported lesion location, they would be directly included in this review. If the lesion location was not explicitly reported in the case, locations on MRI were used. Lesion extension was divided into unilobed lesions, multilobed lesions, and hemispheric lesions. Furthermore, in this review, the occipital or parietal lobes were defined as the posterior regions.

The seizure type (semiology) was determined as follows: (1) cases that explicitly stated the seizure type; (2) if the seizure type was not specified, classification was manually performed based on the International League Against Epilepsy (ILAE) seizure classification in 2017^15^; (3) cases that did not meet the criteria of (1) and (2) were excluded from the analysis. All seizure types throughout the course of epilepsy were recorded. The prevalence of status epilepticus (SE) and the seizure frequency were also recorded. For cognitive and behavioral impairments, the presence of intellectual disability, attention deficit hyperactivity disorder (ADHD), and autism was recorded.

This review collected interictal and ictal EEG data from both intracranial and scalp EEG results. For the interictal EEG results, “circumscribed” was defined as interictal epileptiform discharges within the lesion area based on MRI while “widespread” was the pattern that extended beyond the lesion boundaries.

For MRI results, we analyzed 10 MRI features of MOGHE as previously reported,^16^ including cortical thickening, cortical/subcortical hyperintense T2/FLAIR signal, deep sulcus, power button sign, changes in the morphology of gyri and sulci (ChaMGeS), transmantle sign, cleft-dimple complex, blurring at the gray matter and white matter transition, vascular abnormalities, and diffuse white matter blurring on T2/FLAIR images. MRI data were collected and described based on the frequency of observed MRI manifestations. For FDG-PET results, the presence of hypometabolism and its relationship to the area of MRI lesions or EEG abnormalities were recorded.

The status of *SLC35A2* variants was collected. We also recorded the detection method and depth of sequencing for *SLC35A2*.

Neuropathologic data were collected to establish the diagnosis of MOGHE.^13^ Therefore, oligodendrocyte hyperplasia was present in all included patients. Other neuropathologic features, including the distribution of oligodendrocytes, the location of proliferated oligodendrocytes, myelination, and the presence and distribution of heterotopic neurons, were collected.

We collected data on the surgery types and postoperative outcomes. If the Engel or ILAE classification was not explicitly stated, the prognosis was determined based on prognostic descriptions according to the Engel classification.^17^ To synthesize evidence on different outcome scales, this review used “favorable surgical outcome” for class 1 in the ILAE scale and class I in the Engel scale and “unfavorable surgical outcome” for classes 2–6 in the ILAE scale and classes II-IV in the Engel scale.^17,18^ The therapeutic effects of d-galactose supplementation were also recorded in this review.

### Methodological Quality Assessment

The risk of bias was analyzed with a tool specifically designed to evaluate case reports and case series.^19^ Detailed information on how the assessment was performed can be found in eTable 1.

### Data Analysis

Continuous data were represented by median (range). Pooled proportions and numbers were calculated for categorical variables. The continuous data were compared using the Mann-Whitney *U* test. The categorical data were compared using the Pearson χ^2^ test or Fisher exact test where appropriate.

In the univariate analysis, the χ^2^ test or Fisher exact test was used to compare categorical data; the Mann-Whitney *U* test was used to compare continuous variables between the groups of favorable and unfavorable surgical outcome, unless otherwise specified. Multiple correction was performed for multilevel variables using the Bonferroni method. Variables with *p* < 0.1 in the univariate analysis were subsequently included in a multivariate logistic regression analysis. The eigenvalue criterion was used to test for multicollinearity in the model, and a kappa value <100 was considered indicative of a low degree of multicollinearity.

In the subgroup analyses, we compared patients according to the age at onset (<10 vs ≥ 10 years) or the *SLC35A2* status (*SLC35A2*-positive vs *SLC35A2*-negative), regarding the following variables: sex, age at onset, epilepsy duration, age at surgery, follow-up time, lesion location, a history of ES, myoclonic seizures, tonic seizures, hyperkinetic, diagnosis of Lennox-Gastaut syndrome (LGS) or West syndrome, cognitive comorbidities, interictal EEG and MRI findings, surgery types, and prognosis. A history of behavioral comorbidities, presentations of ictal EEG, FDG-PET, and certain seizure types (tonic-atonic, clonic seizure, akinetic, gelastic, impaired awareness, atypical absence seizure, aphasic, autonomic) and seizure syndromes (Dravet) was not included in subgroup analyses because of insufficient data in subgroups.

To explore the potential impact of the excluded studies, sensitivity analysis was performed by aggregating studies with insufficient individual patient data.

Statistical analysis was performed using Statistical Package for the Social Sciences (SPSS) Statistics version 26 and R version 4.3.1. All hypothesis testing was 2-sided. The threshold for significance was *p* < 0.05.

### Standard Protocol Approvals, Registrations, and Patient Consents

The systematic review was performed according to the Preferred Reporting Items for Systematic Reviews and Meta-Analyses (PRISMA) statement.^20^ The review has been registered and updated on PROSPERO (registration number: CRD42023472978). Institutional review board approval for this review was not required because we only used publicly accessible, published data where informed consent was obtained by each original study.

### Data Availability

All data were derived from resources available in the public domain (eTable 2).

## Results

### Article Selection

**Figure 1:**
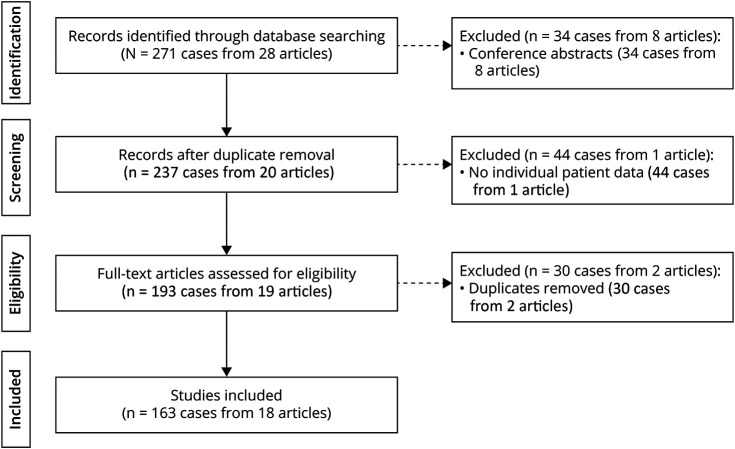
PRISMA Flowchart PRISMA = Preferred Reporting Items for Systematic Review and Meta-Analyses.

After identifying 28 records, we included 18 studies (eTable 2), consisting of 9 case reports and 9 case series.^4-6,9,10,12,21-32^ Excluded were 8 conference abstracts with 34 cases,^33-40^ 1 case series with 44 cases because of lack of individual patient data,^8^ and 2 case series with 30 cases because of duplicate reports.^3,9^ Ultimately, 163 patients with MOGHE were included for further analysis. The search algorithm and flowchart are presented in Figure 1.

### Patient Characteristics

**Figure 2:**
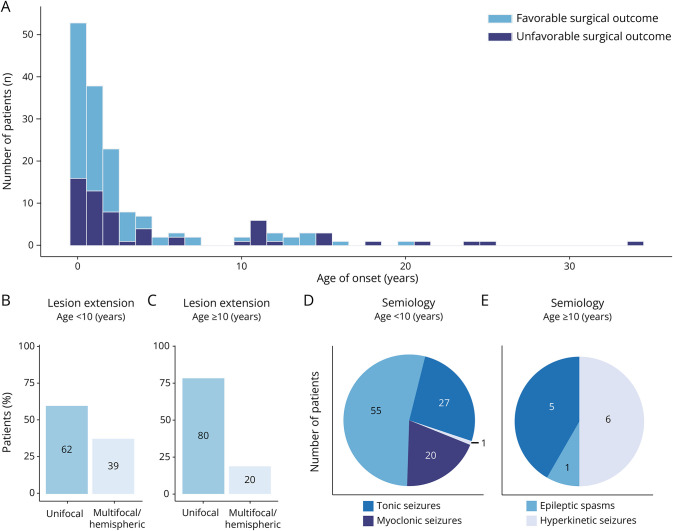
Age at Onset and Phenotypes in Mild Malformation of Cortical Development and Oligodendroglial Hyperplasia The figure 2D needs to be corrected (please see attached figure 2 for the correct version) (A) Patients with MOGHE had a bimodal distribution in age at onset with distinct prognostic profiles. Patients with an onset before 10 years were more likely to reach favorable surgical outcomes than those with an onset age 10 years and older. (B and C) The extent of lesions in patients with MOGHE with an onset age younger than 10 years and 10 years and older. (D and E) Epileptic spasms, tonic seizures, and myoclonic seizures were the predominant semiology in MOGHE with an onset before 10 years while tonic seizures and hyperkinetic seizures were the most common semiology in those with an onset age 10 years and older. MOGHE = mild malformation of cortical development and oligodendroglial hyperplasia.

Detailed data on 163 patients with MOGHE were obtained. All cases (100%) were classified as drug-resistant epilepsy. There was a greater predisposition of male patients to have MOGHE (male: female = 103:60). The median age at onset was 1.2 years (1 day–34 years) with a bimodal distribution (Figure 2). The median epilepsy duration was 3.9 years (4 months–35.1 years). The median age at surgery was 5.4 years (1 year–59.1 years). There was a strong correlation between the age at onset, age at surgery, and epilepsy duration (*p* < 0.001).

### Lesion Distribution

One hundred sixty-three cases reported MRI findings, of which 15 were MRI-negative. The remaining 148 cases were classified into unilobed lesions, multilobed lesions, and hemispheric lesions. Among them, 95 cases (64.2%) were classified as unilobed lesions, 37 (25%) as multilobed lesions, and 16 (10.8%) as hemispheric lesions. Within the unilobed lesion group, 84 cases (88.4%) had lesions in the frontal lobe, 10 (10.5%) in the temporal lobe, and 1 (1.1%) in the parietal lobe. Within the multilobed lesion group, 15 cases (40.5%) involved the frontal lobe, 32 (86.5%) in the temporal lobe, 16 (43.2%) in the occipital lobe, 16 (43.2%) in the parietal lobe, and 14 (37.8%) in the insular lobe. Overall, for patients with focal-onset epilepsies, 99 cases (66.9%) involved the frontal lobe, 42 (28.4%) the temporal lobe, 16 (10.8%) the occipital lobe, 17 (11.5%) the parietal lobe, and 14 (9.5%) the insular lobe.

### Seizure Semiology and Comorbidities

A total of 132 cases with information on seizure types were included in this analysis, with 97 cases reporting seizure frequency. The most common seizure types were ESs (n = 78, 59.1%), tonic seizures (n = 45, 34.1%), and myoclonic seizures (n = 25, 18.9%). Impaired awareness was reported in 27 patients (20.5%), and generalized tonic-clonic attacks were reported in 13 patients (9.8%). The most reported epileptic syndromes were West syndrome (n = 52, 39.4%), LGS (n = 20, 15.2%), and the Dravet syndrome (n = 11, 8.3%). The reported seizure frequency ranged from 0 to several hundred times per day. SE was reported in 10 patients. Notably, 8 patients (66.7%) had a history of SE within one cohort.^29^

Developmental delay was common in MOGHE. Ninety-two patients reported cognitive or behavioral comorbidities. Among them, 83 patients (90.2%) had varying degrees of intellectual disability, 10 (10.9%) had ADHD, and 11 (12.0%) reported autism.

### EEG

Among the 84 cases with scalp EEG during the interictal period, 57 cases (67.9%) reported a “widespread” EEG pattern, 27 (32.1%) reported a “circumscribed” pattern, and 84 (100%) showed epileptiform discharges (spikes, polyspikes, spike-wave complexes). Among the 27 cases with intracranial EEG during the interictal period, 27 (100%) exhibited similar epileptiform discharges including spikes, polyspikes, and spike-wave complexes. Among the 14 cases with scalp EEG during the ictal period, the most common presentation was low-amplitude fast activity at onset in 7 cases (50.0%). Among the 8 cases with intracranial EEG during the ictal period, the most common presentation was low-amplitude fast activity in 5 cases (62.5%) and the other 3 cases (37.5%) demonstrated fast activity at onset.

### Neuroimaging

The most common features of lesions on MRI were cortical/subcortical hyperintense T2/FLAIR signals and blurring at the GM and WM transition. A total of 107 cases were included, with MRI findings reported as follows: blurring at the gray matter and white matter transition in 74 cases (69.2%), cortical/subcortical hyperintense T2/FLAIR signal in 60 (56.1%), cortical thickening in 38 (35.5%), and ChaMGeS in 24 (22.4%). Eight cases (7.5%) exhibited deep sulcus and diffuse white matter blurring on T2/FLAIR images. The cleft-dimple complex was identified in 6 cases (5.6%) while the transmantle sign was seen in 3 (2.8%). Furthermore, a power button sign was reported in 1 patient (0.9%). Vascular abnormalities were not detected in any of the cases.

A total of 74 cases were included in the analysis of FDG-PET findings. Of these, hypometabolism was reported in 72 cases (97.3%). Among the 72 cases with reported hypometabolism, 33 cases (45.8%) indicated that the hypometabolic region corresponded to the detected lesions on MRI and 18 cases (25%) showed hypometabolism regions extending beyond the detected lesions. The remaining 21 cases (29.2%) did not report associations with MRI lesions. In an 11-patient cohort that reported the relationship between regions with PET abnormalities and ictal EEG onset, 9 cases (13.2%) showed concordance between the hypometabolism location and the ictal EEG region.^4^ In another cohort that reported the relationship between regions with PET abnormalities and the epileptogenic zone, 9 cases (100%) showed concordance between the 2 regions.^29^

### Histopathology

The diagnostic hallmarks of MOGHE include oligodendrocyte hyperplasia on pathology, often with heterotopic neurons in the white matter.^13^ The proliferated oligodendrocytes had a multifocal distribution with patchy infiltration, in clusters, or had a focally laminar organization. The proliferated oligodendrocytes were mainly in the white matter including the gray-white matter junction. Hypomyelination was reported in patients with MOGHE, adjacent to areas of oligodendrocyte hyperplasia. Heterotopic neurons, an additional pathologic feature of MOGHE, were primarily observed in the white matter.

### Treatment and Prognosis

Major treatments of MOGHE included ASMs and surgeries. A total of 160 cases were enrolled for surgical treatment and prognosis, among which 101 cases (63.1%) had a favorable surgical outcome. One-hundred 32 patients reported follow-up durations, and 87 (65.9%) achieved favorable surgical outcomes with a median follow-up of 2 years (0.25 years–7 years). Among the 19 cases with a second follow-up, 4 cases (21.1%) experienced recurrence. Recent studies found a significant improvement (seizure frequency decreased by 50%) in 4 of 6 patients who received postoperative d-galactose supplementation.^12^

Patients with an earlier onset (*p* = 0.028), those with an earlier age at surgery (*p* = 0.006), and those having lobar disconnection (*p* = 0.001) and a circumscribed pattern of interictal EEG (*p* = 0.008) were associated with a favorable surgical outcome. No statistically significant differences were found in sex, epilepsy duration, follow-up time, lesion, semiology, epileptic syndromes, intellectual levels, and *SLC35A2* variants between MOGHE with favorable and unfavorable surgical outcomes (Table 1). However, the age at onset and the age at surgery were highly correlated (*p* < 0.001). The surgery types and interictal EEG patterns were also highly correlated with the age at onset (*p* < 0.001). Therefore, multivariate regression was not performed because of the high multicollinearity of the variables.

**Table 1:** Clinical Characteristics and Outcomes in Mild Malformation of Cortical Development and Oligodendroglial Hyperplasia

	Total	Surgical outcomes		*p* Value
		Favorable	Unfavorable	
Sex, n (%)				
Male	101 (63.1)	66 (65.3)	35 (34.7)	0.446^a^
Female	59 (36.9)	35 (59.3)	24 (40.7)	
Age at onset, median (range), y	1.21 (0–34)	1.1 (0–20)	1.58 (0.25–34)	0.028^d^
Duration, median (range), y	3.8 (0.33–35.1)	3.6 (0.33–21)	6 (0.67–35.1)	0.053^d^
Age at surgery, median (range), y	5.4 (1–59.1)	4.9 (1–33)	9.58 (1.33–59.1)	0.006^d^
Follow-up, median (range), y	2 (0.25–7)	2 (0.25–7)	2 (0.25–7)	0.177^d^
Lesion extension, n (%)				
Unilobar	92 (63.4)	58 (63)	34 (37)	0.894^a^
Multilobar/hemispheric	53 (36.6)	34 (64.2)	19 (35.8)	
Lesion distribution, n (%)				
Frontal lobe	96 (66.2)	62 (67.4)	34 (64.2)	0.691^a^
Temporal lobe	42 (29)	27 (29.3)	15 (28.3)	0.894^a^
Insular lobe	14 (9.7)	10 (10.9)	4 (7.5)	0.514^a^
Posterior regions	22 (15.2)	16 (17.4)	6 (11.3)	0.326^a^
Semiology, n (%)				
Epileptic spasms	56 (59.6)	39 (60.9)	17 (56.7)	0.694^a^
Myoclonic seizures	19 (20.2)	12 (18.8)	7 (23.3)	0.606^a^
Tonic seizures	31 (33)	19 (29.7)	12 (40.0)	0.322^a^
Hyperkinetic seizures	7 (7.4)	5 (7.8)	2 (6.7)	>0.999^b^
Epileptic syndromes, n (%)				
Lennox-Gastaut syndrome	20 (18.0)	13 (16.7)	7 (21.2)	0.655^a^
West syndrome	52 (46.8)	37 (47.4)	15 (45.5)	0.848^a^
Dravet syndrome	2 (1.8)	1 (1.3)	1 (3.0)	0.508^b^
Intellectual level, n (%)				
Normal	8 (13.8)	3 (37.5)	5 (62.5)	0.681^b^
Below average	50 (86.2)	14 (28)	36 (72)	
Interictal EEG discharges, n (%)				
Circumscribed	19 (30.2)	17 (89.5)	2 (10.5)	0.008^a^
Widespread	44 (69.8)	24 (54.5)	20 (45.5)	
Surgical types, n (%)				
Hemispherectomy	13 (8.2)	10 (76.9)	3 (23.1)	0.001^bc^
Disconnections	23 (14.5)	22 (95.7)	1 (4.3)	
Sublobectomy/lesionectomy	121 (76.1)	68 (53.2)	53 (46.8)	
*SLC35A2* positivity, n (%)				
Positive	44 (73.3)	31 (70.5)	13 (29.5)	0.302^a^
Negative	16 (26.7)	9 (56.3)	7 (43.8)	

a Pearson χ^2^ test.

b Fisher exact test.

c The results of the Bonferroni test indicated that there was a statistically significant difference between disconnection and sublobectomy/lesionectomy at a significance level of 0.05.

d Mann-Whitney test.

### Age-Dependent Phenotypes

Significant differences in phenotypes were observed between young children (onset before 10 years) and postpubertal children and adults with MOGHE (onset age 10 years and older) (Table 2). Among children with an onset before 10 years, there were more male patients than female patients (male: female = 89:47). The younger group also had a shorter epilepsy duration (*p* < 0.001). In the semiology analysis, 95 cases from 14 studies were included, given the availability of data on individual symptoms, and 110 cases from 15 studies were included for the epileptic syndrome analysis. ESs were more common in those with an early onset (*p* < 0.001) while hyperkinetic attacks were more common in the older cohorts (*p* < 0.001). All cases with myoclonic seizures were reported in patients with an onset before 10 years. The West syndrome was more commonly reported in those with an early onset (*p* < 0.001).

**Table 2:** Age-Dependent Phenotypes in Mild Malformation of Cortical Development and Oligodendroglial Hyperplasia

	Total	Onset <10 y	Onset ≥10 y	*p* Value
Sex, n (%)				
Male	102 (63.4)	89 (65.4)	13 (52)	0.2^a^
Female	59 (36.6)	47 (34.6)	12 (48)	
Duration, median (range), y	3.9 (0.33–35.1)	3.6 (0.33–25.7)	12.5 (3–35.1)	<0.001^c^
Follow-up, median (range), y	2 (0.17–7)	2 (0.17–7)	2.8 (0.5–7)	0.224^c^
Lesion extension, n (%)				0.078^a^
Unilobar	95 (64.6)	75 (61.5)	20 (80)	
Multilobar/hemispheric	52 (35.4)	47 (38.5)	5 (20)	
Lesion distribution, n (%)				
Frontal lobe	99 (67.3)	82 (67.2)	17 (68)	0.939^a^
Temporal lobe	42 (28.6)	32 (26.2)	10 (40)	0.165^a^
Insular lobe	14 (9.5)	13 (10.7)	1 (4)	0.465^b^
Posterior regions	22 (15)	20 (16.4)	2 (8)	0.37^b^
Semiology, n (%)				
Epileptic spasms	56 (58.9)	55 (66.3)	1 (8.3)	<0.001^b^
Myoclonic seizures	20 (21.1)	20 (24.1)	0 (0)	0.065^b^
Tonic seizures	32 (33.7)	27 (32.5)	5 (41.7)	0.53^b^
Hyperkinetic seizures	7 (7.4)	1 (1.2)	6 (50)	<0.001^b^
Epileptic syndromes, n (%)				
West syndrome	51 (46.4)	51 (52.0)	0 (0)	<0.001^b^
Lennox-Gastaut syndrome	20 (18.2)	20 (20.4)	0 (0)	0.119^b^
Dravet syndrome	2 (1.8)	2 (2.0)	0 (0)	>0.999^b^
Intellectual level, n (%)				
Normal	9 (9.8)	5 (5.9)	4 (57.1)	0.001^b^
Below average	83 (90.2)	80 (94.1)	3 (42.9)	
MRI features, n (%)				
Cortical thickening	38 (35.5)	32 (35.6)	6 (35.3)	0.984^a^
Cortical/subcortical hyperintensities	60 (56.1)	51 (56.7)	9 (52.9)	0.777^a^
ChaMGeS	24 (22.4)	18 (20)	6 (35.3)	0.184^b^
Blurring at the gray and white matter transition	74 (69.2)	60 (66.7)	14 (82.4)	0.199^a^
Interictal EEG discharges, n (%)				
Circumscribed	20 (31.3)	20 (40.8)	0 (0)	0.003^b^
Widespread	44 (68.8)	29 (59.2)	15 (100)	
Surgical types, n (%)				
Hemispherectomy	13 (8.3)	13 (9.8)	0 (0)	0.009^b^
Disconnections	23 (14.6)	23 (17.3)	0 (0)	
Sublobectomy/lesionectomy	121 (77.1)	97 (72.9)	24 (100)	
Surgical outcomes, n (%)				0.011^a^
Favorable	99 (62.7)	89 (66.9)	10 (40)	
Unfavorable	59 (37.3)	44 (33.1)	15 (60)	

a Pearson χ^2^ test.

b Fisher exact test.

c Mann-Whitney test.

In addition, intellectual disabilities were significantly more common (*p* = 0.001), with a slightly higher likelihood of multilobed or hemispheric lesions on MRI (*p* = 0.078). The younger onset patients were more likely to undergo extensive resections (hemispherectomy/disconnections) (*p* = 0.009) and had a higher chance of favorable surgical outcomes (*p* = 0.011) (Table 2).

Previous studies reported a conversion of MRI patterns between the ages of 3 and 6 years. Conversions from subtype I (laminar subcortical T2/FLAIR hyperintensities at the corticomedullary junction) to subtype II (reduced corticomedullary differentiation) were observed with an increasing age in 5 patients.^6,9^

### *SLC35A2*-Associated Phenotypes

The analysis included 62 cases from 5 studies reporting the results of gene panel sequencing. Four studies (61 cases) used deep sequencing, including 1 with a mean read depth of 150 (12 cases), 1 with >100,000x read depth (10 cases), and another (37 cases) with a mean sequencing coverage of 1,161x.^4,12,26^ In the other study (2 cases), matched blood and surgical samples were sent to 5 genetic laboratories, where those with a read depth greater than 1,000 were able to detect the *SLC35A2* variants.^10^ One case report used Sanger sequencing for the *SLC35A2* gene.^32^

Among the 62 cases, 45 (72.6%) were *SLC35A2*-positive. Notably, 58 patients (17 *SLC35A2*-negative and 41 *SLC35A2*-positive patients) had an onset before 10 years. Only 2 patients (*SLC35A2*-positive) had an onset age 10 years and older. The age at onset in the other 2 *SLC35A2*-positive patients were not reported.^4^ To determine the potential genotype-phenotype association, we compared the clinical phenotypes and surgical outcomes between *SLC35A2*-positive (n = 45) and *SLC35A2*-negative (n = 17) patients, as shown in eTable 3. Patients with *SLC35A2* variants had a higher proportion of LGS diagnosis.

### Sensitivity Analysis

The sensitivity analysis is summarized in eTable 4. Inclusion of the study by Barba et al.^8^—which was removed in the final analysis because of a lack of individual patient data—yielded similar results including sex, onset, surgery age, lesion distributions, semiology features, surgical procedures, and prognosis.

### Assessment of Methodological Quality

The results of the risk-of-bias assessment are provided in eTable 1. Overall, 15 articles (83.3%) had a low risk of bias and 3 articles (16.7%) had a moderate risk of bias.

## Discussion

MOGHE is an increasingly recognized yet rare etiology of drug-resistant epilepsy. It is characterized by oligodendroglia hyperplasia on pathology, although the clinical spectrum remains diverse and unclear. We systematically analyzed the demographic and clinical features, EEG, neuroimaging, histopathologic findings, treatment, and prognosis of MOGHE based on 163 patients from 18 published case reports or case series. We also reported factors associated with the surgical prognosis in patients with MOGHE. The comprehensive analysis delineated an objective picture of the rare disease and revealed an age-dependent and genotype-associated phenotype in patients with MOGHE.

MOGHE was characterized by drug-resistant epilepsy and a widespread seizure network. It predominantly occurred in children. Nearly half of the MOGHE cases started with ESs as the initial presentation and were often accompanied by cognitive impairments. The lesion most frequently involved the frontal lobe. Interictal EEG predominantly showed widespread spikes, and low-voltage fast activity was the most frequent EEG presentation at seizure onset. On MRI, typical features of MOGHE included blurred gray-white matter transition and cortical/subcortical hyperintense T2/FLAIR signals, with approximately one-third of cases encompassing multiple lobes. A single-center cohort reported that all 12 patients had ChaMGeS, which may serve as diagnostic biomarkers of MOGHE.^29^ In addition, cortical dimples may aid in the localization of MOGHE lesions.^29^ FDG-PET revealed widespread interictal hypometabolism often extending beyond the MRI lesion. Approximately 50% of patients tested positive for *SLC35A2* variants.^4,12^ Altogether, the clinical and ancillary findings suggest a widespread seizure network in MOGHE.

However, the overall surgical prognosis of MOGHE was relatively favorable compared with other subtypes of cortical malformations.^41^ An earlier onset, an earlier age at surgery, surgery types of lobar disconnection, and a circumscribed pattern of interictal EEG were highly associated with a favorable outcome. The strong correlation between the extent of surgeries and the surgical outcome was consistent with previous single-center studies.^5,6^ These findings indicate that resection surgeries might be insufficient to remove the epileptogenic zone, and extensive procedures should be considered in presurgical evaluations.

Of interest we noticed strikingly distinct phenotypes between patients with MOGHE with a younger (onset before 10 years) and an older (onset age 10 years and older) onset. Young children predominantly presented with ESs were more likely to have intellectual disabilities, multilobed lesions, and a shorter epilepsy duration. They were more likely to undergo more destructive surgical procedures. The prognosis was substantially better in this group, with 66.9% reaching favorable surgical outcomes, compared with only 40.0% among postpubertal children and adults. The better outcome, despite a seemingly extensive lesion, may be attributed to the shorter epilepsy duration and the higher likelihood of using hemispherectomy or disconnections as the preferred surgical procedure. Of interest the age-dependent characteristics in MOGHE, although unique in the MCD entity, were shared with other white matter or oligodendroglia-associated diseases,^42^ including myelin oligodendrocyte glycoprotein antibody-associated disease (MOGAD) and multiple sclerosis.^43^ The similarity suggests a potential link between MOGHE and the underlying myelination processes during brain development, which normally rapidly advances in the first 5 years of life.^44^ In the cases we gathered, 95% of those with an early onset had an onset before 5 years, aligning with the developmental time frame of myelin. This period is characterized by susceptibility of immature white matter to injuries, leading to a broader range of anatomical lesions. In addition, the frontal and temporal lobes undergo longer periods of myelination compared with other regions of the developing brain, making them susceptible to developmental disturbances.^45^

Another observation was the strong association between *SLC35A2* variants and MOGHE. *SLC35A2* mosaicism was found in at least 50% of patients with histologic diagnosis of MOGHE.^4,12^ It should be noted that the selection of patients for genetic testing, inadequate sampling, inappropriate testing methods, inadequate sequencing depth, and a low variant load may lead to an underestimation of the proportion of *SLC35A2* mosaicism in MOGHE. In our study, almost all the patients with *SLC35A2* variants had an onset before 10 years. Among them, *SLC35A2*-positive patients were more likely to present with LGS. In line with the observed genotype-phenotype association, cumulating evidence suggests the pathogenicity of *SLC35A2* in the development of epilepsy. The density of interictal EEG spikes was positively correlated with the variant allele burden of *SLC35A2*.^46^ Patients with a lower variant burden had better cognitive improvement after surgery.^8^ Pathogenic *SLC35A2* variants are associated with defect UDP-galactose transport into the Golgi apparatus, and preliminary data suggested the efficacy of postoperative supplementation with d-galactose in improving the outcome of patients with MOGHE.^4,12^ Nevertheless, direct evidence on the pathogenic role of *SLC35A2* is lacking. Experimental studies with *SLC35A2* knockdown or knockout may shed further light on its role in neuronal migration, myelin formation, and the development of epilepsy.

Certain limitations should be considered to properly interpret the results of this systematic review. First, data extracted from published reports were subject to the inherent selection bias of the report itself. Certain symptoms and ancillary findings can be underestimated because they were not observed by the reporting author or not reported because of the different aims of the study. Second, given the relatively short history of MOGHE, the follow-up time was variable and shorter, with a median follow-up time of 2 years. This meant that important information on the long-term disease course after surgery was not available, which could have led to biased prognostic and recurrence outcomes due to insufficient follow-up time.

In future research, prospective studies with longer follow-up periods should be considered. In addition, d-galactose supplementation emerges as a safe and feasible treatment option for MOGHE. Further investigations are needed to confirm its efficacy, identify biomarkers of response, and elucidate the underlying therapeutic mechanisms.

The systematic review provides a comprehensive understanding of MOGHE, a newly described entity of mild MCD. The analysis highlighted significant findings, including an age-dependent phenotype, *SLC35A2* variant–related features, and factors predictive of surgical outcomes in patients with MOGHE. These findings contribute to an early recognition, accurate diagnosis, and prompt management of MOGHE. The study emphasizes the importance of considering age-related phenotypes and genetic factors in the clinical evaluation of patients with MOGHE.

## Glossary

ADHD: attention deficit hyperactivity disorder
ASM: antiseizure medication
ChaMGeS: changes in the morphology of gyri and sulci
ES: epileptic spasm
FDG-PET: Fluorodeoxyglucose-Positron Emission Tomography
FLAIR: fluid attenuated inversion recovery
GW: grey matter
ILAE: International League Against Epilepsy
LGS: Lennox-Gastaut syndrome
MCD: mild malformation of cortical development
MOGAD: myelin oligodendrocyte glycoprotein antibody-associated disease
MOGHE: mild malformation of cortical development with oligodendroglial hyperplasia and epilepsy
PRISMA: Preferred Reporting Items for Systematic Reviews and Meta-Analyses
SE: status epilepticus
SPSS: Statistical Package for the Social Sciences
WM: white matter
